# Enhanced Recovery After Cardiac Surgery for Minimally Invasive Valve Surgery: A Systematic Review of Key Elements and Advancements

**DOI:** 10.3390/medicina61030495

**Published:** 2025-03-13

**Authors:** Simon Goecke, Leonard Pitts, Martina Dini, Matteo Montagner, Leonhard Wert, Serdar Akansel, Markus Kofler, Christian Stoppe, Sascha Ott, Stephan Jacobs, Benjamin O’Brien, Volkmar Falk, Matthias Hommel, Jörg Kempfert

**Affiliations:** 1 Department of Cardiothoracic and Vascular Surgery, Deutsches Herzzentrum der Charité (DHZC), Augustenburger Platz 1, 13353 Berlin, Germany; leonard.pitts@dhzc-charite.de (L.P.); martina.dini@dhzc-charite.de (M.D.); matteo.montagner@dhzc-charite.de (M.M.); leonhard.wert@dhzc-charite.de (L.W.); serdar.akansel@dhzc-charite.de (S.A.); markus.kofler@dhzc-charite.de (M.K.); stephan.jacobs@dhzc-charite.de (S.J.); volkmar.falk@dhzc-charite.de (V.F.); joerg.kempfert@dhzc-charite.de (J.K.); 2 Charité—Universitätsmedizin Berlin, Corporate Member of Freie Universität Berlin and Humboldt-Universität zu Berlin, Charitéplatz 1, 10117 Berlin, Germany; christian.stoppe@dhzc-charite.de (C.S.); sascha.ott@dhzc-charite.de (S.O.); ben.obrien@dhzc-charite.de (B.O.); matthias.hommel@dhzc-charite.de (M.H.); 3 DZHK (German Centre for Cardiovascular Research), Partner Site Berlin, 10785 Berlin, Germany; 4 Department of Cardiac Anesthesiology and Intensive Care Medicine, Deutsches Herzzentrum der Charité (DHZC), Augustenburger Platz 1, 13353 Berlin, Germany; 5 Department of Anaesthesiology, Intensive Care, Emergency and Pain Medicine, University Hospital Würzburg, Oberdürrbacher Str. 6, 97080 Würzburg, Germany; 6 Translational Cardiovascular Technologies, Institute of Translational Medicine, Department of Health Sciences and Technology, Swiss Federal Institute of Technology (ETH), 8092 Zurich, Switzerland

**Keywords:** enhanced recovery after surgery, minimally invasive valve surgery, cardiac surgery, length of stay, mitral valve surgery, aortic valve surgery

## Abstract

*Background and Objectives*: Minimally invasive valve surgery (MIVS), integrated within enhanced recovery after surgery (ERAS) programs, is a pivotal advancement in modern cardiac surgery, aiming to reduce perioperative morbidity and accelerate recovery. This systematic review analyzes the integration of ERAS components into MIVS programs and evaluates their impact on perioperative outcomes and patient recovery. *Materials and Methods*: A systematic search of PubMed/Medline, conducted according to PRISMA (Preferred Reporting Items for Systematic Reviews and Meta-Analyses) guidelines, identified studies on ERAS in MIVS patients. Coronary and robotic surgery were excluded to prioritize widely adopted minimally invasive valve methods. Studies were included if they applied ERAS protocols primarily to MIVS patients, with at least five participants per study. Data on study characteristics, ERAS components, and patient outcomes were extracted for analysis. *Results*: Eight studies met the inclusion criteria, encompassing 1287 MIVS patients (842 ERAS, 445 non-ERAS). ERAS protocols in MIVS were heterogeneous, with studies implementing 9 to 18 of 24 ERAS measures recommended by the ERAS consensus guideline, reflecting local hospital practices and resource availability. Common elements include patient education and multidisciplinary teams, early extubation followed by mobilization, multimodal opioid-sparing pain management, and timely removal of invasive lines. Despite protocol variability, these programs were associated with reduced morbidity, shorter hospital stays (intensive care unit-stay reductions of 4–20 h to complete omission, and total length of stay by ≥1 day), and cost savings of up to EUR 1909.8 per patient without compromising safety. *Conclusions*: ERAS protocols and MIVS synergistically enhance recovery and reduce the length of hospital stay. Standardizing ERAS protocols for MVS could amplify these benefits and broaden adoption.

## 1. Introduction

Minimally invasive valve surgery (MIVS) of the aortic and mitral valves, both first successfully performed in 1996, represents a significant leap in cardiac surgery by avoiding full median sternotomy [1,2]. Compared to traditional open-sheart surgery by median sternotomy, minimally invasive techniques have been very effective in reducing surgical trauma, recovery times, and complication rates for several cardiac surgery procedures [3,4]. Diagnosis is based on clinical presentation and imaging (primarily echocardiography), while the surgery decision is made by the heart team following the respective guidelines for the management of valvular heart disease and patient goals [5]. Contraindications arise from concomitant surgery, anatomical incompatibilities, and limited surgical access, often identified via computed tomography [6,7].

Complementarily, the implementation of enhanced recovery after surgery (ERAS) protocols in various surgical specialties, including cardiac surgery, aims to improve outcomes and perioperative care [8]. These protocols were initially predominantly based on fast-track approaches to expedite extubation. They were subsequently extended to comprehensive, multidisciplinary approaches designed to optimize perioperative care. ERAS programs aim to reduce patient morbidity, ultimately leading to reduced hospital length of stay (LOS) without compromising safety [9].

The latest framework for ERAS protocols in cardiac surgery, a joint consensus statement, was published in 2024 by the ERAS Cardiac Society, ERAS International Society, and The Society of Thoracic Surgeons. This statement defines 24 ERAS measures grouped into five categories based on their application phase during the perioperative hospital stay [10].

Although several studies have integrated ERAS protocols in MIVS, including the broad spectrum of established ERAS components, each protocol follows an individual approach. This systematic review aims to evaluate ERAS protocols tailored to MIVS, dissecting their individual components. Additionally, innovative approaches and potential future modifications to these protocols are discussed. The objective is to identify key elements and strategies to advance available ERAS cardiac protocols to provide optimal results for patients undergoing MIVS.

## 2. Materials and Methods

This systematic review was conducted following the Preferred Reporting Items for Systematic Reviews and Meta-Analyses (PRISMA). It focused on ERAS trials in MIVS patients published in the MED-LINE/PubMed database. For these, the keywords “ERAS” were combined with “minimally invasive valve surgery” OR “minimally invasive mitral surgery” OR “minimally invasive aortic surgery”. The MED-LINE search was conducted using the following query: ((Fast-track) OR (Fast Track) OR (ERAS) OR (ERACS) OR (enhanced recovery after cardiac surgery) OR (enhanced recovery after surgery)) AND ((mini* invasive heart valve surgery) OR (mini* invasive cardiac surgery) OR (mini* invasive mitral surgery) OR (mini* invasive aortic surgery) OR (mini* invasive valve surgery)). The final search was executed on 15 December 2024.

Titles, abstracts, and full texts were reviewed, with exclusions detailed in the PRISMA flowchart. Studies were included if they met the following criteria: they involved a cohort of 5 or more patients undergoing MIVS, were published in the English language, and reported at least one of the following outcomes: safety, feasibility, complications, morbidity, mortality, or efficacy of the corresponding ERAS program.

Papers on full median sternotomy, coronary surgery, and robotic surgery were excluded to ensure a focus on standardized minimally invasive valve surgery approaches as the baseline for the ERAS protocols. Full sternotomy was excluded due to its invasive nature, coronary surgery due to its differing patient population, and robotic surgery to avoid conflating this developing field with established minimally invasive techniques. Reviews, letters, case reports, editorials, meeting abstracts, replies, and papers not meeting inclusion criteria were excluded.

The review process, including initial screening, eligibility assessment, and data extraction, was conducted by S.G. and independently validated by L.P.

Data collected encompassed study characteristics (e.g., study period, sample size, type of procedures), primary and secondary outcomes (e.g., morbidity, LOS, and complications), and key ERAS elements. Whenever possible, statistical values such as confidence intervals and *p*-values were extracted from the original studies. According to the consensus statement, ERAS elements were categorized into five groups: general, preoperative, intraoperative, postoperative, and multiphase measures. While patient characteristics, preoperative risk factors, and intraoperative values were recorded for completeness, our analysis focused on ERAS measures and postoperative outcomes to align with the study’s objectives. The extracted data are presented as mean ± standard deviation, median with interquartile ranges (Q1–Q3), or as percentages, depending on the type of variable analyzed. These were presented whenever reported in the original studies.

## 3. Results

### 3.1. Study Selection

**Figure 1:**
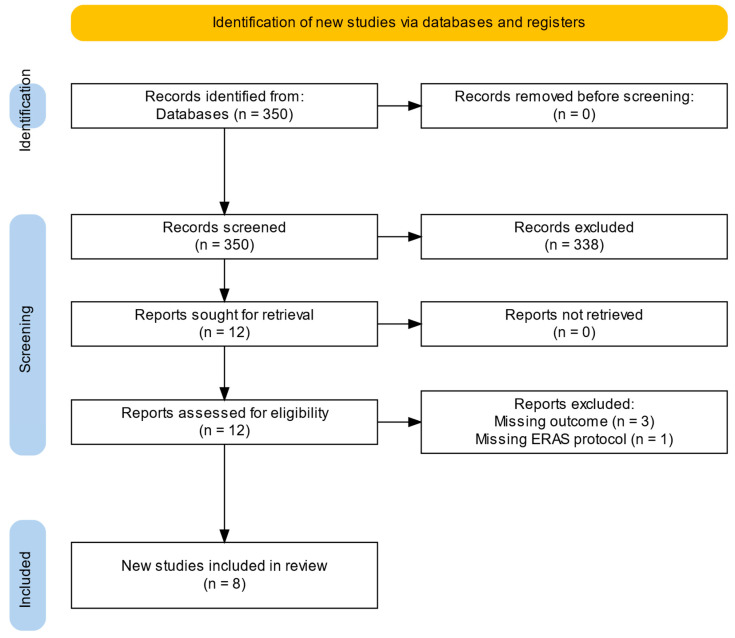
PRISMA flow diagram showing study selection. Created using the PRISMA2020 Shiny app [23]. Abbreviations: ERAS = Enhanced Recovery After Surgery.

Out of 350 publications identified through the literature search, titles and abstracts were screened for relevance, resulting in the full-text assessment of 12 publications (Figure 1). In total, eight publications fulfilled the inclusion criteria and were included in this systematic review [11,12,13,14,15,16,17,18] (Table 1). Four publications were excluded after full-text screening as they did not meet the inclusion criteria. They were omitted due to missing outcome data (*n* = 3) [19,20,21], and the absence of an ERAS protocol (*n* = 1) [22]. These studies reported only ERAS concepts, short abstracts, or qualitative analyses without relevant outcome data for analysis.

The studies comprised a total of 1287 patients, with 842 following ERAS protocols and 445 as a control group. The study types varied, with three observational cohort studies [11,13,15], two propensity-matched analyses [14,18], and three pilot studies [12,16,17]. Five of these studies were conducted in Germany, with contributions from research groups based in Hamburg [12,13,15], Augsburg [16], and Berlin [18]. The other studies were published by teams from Bordeaux, France [11]; Ancona, Italy [14]; and İzmir, Turkey [17].

The studies were analyzed following the most recent recommendations of the joint consensus statement and are listed in Table 2. They are structured in general (rows 2–4), preoperative (rows 4–6), intraoperative (rows 7–14), postoperative (rows 15–18), and multiphase (rows 19–25) elements.

### 3.2. General Elements

General elements are presented in Table 2, rows 2–4.

#### 3.2.1. Shared Decision Making, Patient Engagement, and Education

Patient education and engagement are central components of ERAS protocols, but their implementation showed high variance across the analyzed studies. Zaouter et al. emphasized preoperative education through interdisciplinary meetings with specialists, supported by a booklet and video detailing the ERAS protocol [11]. Kubitz et al., Petersen et al., and Gebauer et al. organized preoperative meetings 2–3 weeks before surgery with multidisciplinary teams to educate patients and introduce physiotherapy exercises and nutrition plans [12,13,15]. They focused on clear communication about roles and expectations to empower patients to actively participate in their care and align with the ERAS protocol [11]. Ertugay et al. provided patients with detailed information about the operative course [17], while Pitts et al. educated patients on the perioperative process of the ERAS program [18].

Emphasis on patient education lays the groundwork for shared decision making and engagement, which enhances satisfaction [21].

#### 3.2.2. Establishment of a Multidisciplinary Team (MDT)

Building on the established heart team structure, a multidisciplinary team is another cornerstone of ERAS protocols [10,24]. It provides a comprehensive medical therapy for each treatment aspect, addressing patients’ risk factors and perioperative challenges [10,21]. While Zaouter et al. did not specify the team composition [11], the studies by Kubitz et al., Petersen et al., and Gebauer et al. included cardiac surgeons, anesthetists, perfusionists, psychologists, and physiotherapists in their ERAS teams [12,13,15]. Berretta et al. expanded this by including nurses [14]. Stock et al. and Ertugay et al. incorporated ERAS nurses into their MDT to streamline the patients’ perioperative stay [16,17]. Ertugay et al. further incorporated dietitians alongside their ERAS team [17]. Pitts et al. implemented a multi-professional heart team performing ERAS safety checkpoints at set time points, including a late evening surgical visit and a midnight check by the intensive care unit (ICU) team [18]. Although the composition of MDTs varied across studies, with larger teams including ERAS coordinators, dietitians, and psychotherapists, no study reported any impact of team size on patient management or organizational effectiveness. Ultimately, ERAS teams adapt to institutional resources, reflecting unique priorities and capabilities [25].

The ERAS coordinator, whose role is increasingly recognized as essential for successful protocol implementation, is critical as an addition to this multidisciplinary approach [25]. The coordinator facilitates interdisciplinary collaboration, audits adherence to ERAS principles, and addresses logistical challenges [26,27]. With the support of the MDT, he guides patients through the perioperative pathway. The integration of such coordinators is particularly relevant in cardiac surgery to bridge gaps between multiple disciplines and ensure the detailed coordination required for successful outcomes. In Ertugay’s surgeon-led program, three ERAS nurses managed the perioperative stay, focusing on patient evaluation, education, and treatments [17]. Pitts et al. relied on an ERAS coordinator to lead implementation, assess patients, and address challenges, ensuring seamless perioperative processes [18].

#### 3.2.3. Compliance and Outcomes Auditing

None of the included studies measured ERAS protocol compliance, for example, adherence to smoking cessation or prehabilitation measures. Several studies made adjustments to their ERAS protocols, reflecting their ongoing auditing. Kubitz et al. modified their anesthesia approach by moving to a continuous propofol infusion following severe postoperative nausea and vomiting (PONV) in the first 20 participants [12]. The pilot studies of Kubitz et al., Stock et al., and Ertugay et al. can be seen as active forms of outcome auditing, as they share their developing ERAS strategies and outcome data [12,16,17]. Ongoing ERAS protocols have now integrated qualitative evaluations of patients, which are anticipated to foster sustainable improvements in these protocols [19,21]. Regular and standardized ERAS team meetings may be implemented to improve patient adherence through auditing [28].

### 3.3. Preoperative Process Measures

Preoperative elements are presented in Table 2, rows 5–7.

#### 3.3.1. Preoperative Screening and Risk Assessment

Preoperative screening is routinely utilized to assess the multimorbidity of patients before surgery. This typically includes patients in need of redo surgery or those with significant comorbidities such as advanced age, chronic kidney disease, or history of stroke [11,12,13,14,15,16,17,18]. Further, most studies checked for frailty and malnutrition [11,12,13,14,15,17]. The patient cohorts in these studies therefore represent a healthier group of cardiac surgery patients, omitting approximately one-third of cardiac surgery patients classified as frail or prefrail [29].

Frailty assessment is especially crucial in patients scheduled for ERAS protocols in MIVS, as frail patients have lower functional reserves and face higher perioperative risks, which poses a challenge to a successful ERAS implementation [27,30]. The European Association for Cardio-Thoracic Surgery (EACTS) and the European Association of Preventive Cardiology (EAPC) recommend initial screening using the Clinical Frailty Scale with a threshold of ≥4 and a gait speed < 0.8 m/s. Patients meeting one of these criteria are classified as frail [31]. It strongly predicts both short- and long-term mortality and may require tailored ERAS approaches [27,31]. Frail patients are subject to significantly higher perioperative complications and up to twice the operative mortality risk [29]. Evidence from non-cardiac ERAS programs further suggests that frail patients may have a prolonged LOS and more frequent readmissions and postoperative emergency department visits [32]. Therefore, frail patients may require adapted strategies regarding ERAS elements like early tube removal (see Section 3.7.2) or selection of anticoagulation (see Section 3.8.5), as their higher complication risk may necessitate a more conservative approach.

Notably, after implementing a prehabilitation regimen (see Section 3.3.2), Ertugay et al. propose to include frail or malnourished patients [17].

#### 3.3.2. Prehabilitation

Patients excluded due to multimorbidity, though likely to benefit from prehabilitation programs, may not be fit enough to endure the ambitious pre- and postoperative physiotherapy required by ERAS protocols [15,29,30,33]. Prehabilitation programs varied across the analyzed studies, while evidence for their effectiveness remains modest, with the 2024 Consensus Statement highlighting low-quality evidence [10]. Zaouter et al. focused on tailored nutritional interventions when necessary [11]. Kubitz et al. and Petersen et al. included daily preoperative exercises for 2–3 weeks and nutritional optimization through energy- and carbohydrate-rich diets [12,13]. Stock et al. described prehabilitation as part of an interdisciplinary preoperative clinic visit, though further details were not specified [16]. Ertugay et al. plan on implementing a prehabilitation protocol in the future to also include an older and more frail population in future trials [17].

#### 3.3.3. Limiting Nil per Os Status

Historically, the 2019 ERAS guidelines recommended preoperative carbohydrate drinks, which were included in protocols such as those by Kubitz et al. [12]. However, recent meta-analyses show these interventions yield inconclusive benefits, with water or placebo providing equivalent outcomes [8,34,35,36]. Therefore, current guidelines emphasize limiting nil per os by abstaining from solid meals for 6 to 8 h and clear liquids for 2 h [10].

### 3.4. Intraoperative Process Measures

Intraoperative elements are presented in Table 2, rows 7–14.

#### 3.4.1. Transesophageal Echocardiography

Transesophageal echocardiography (TEE) plays a crucial role in MIVS, offering detailed visualization that outperforms standard preoperative transthoracic echocardiography. For example, in right anterolateral thoracotomy (RALT) or trans-axillary access, it guides the placement of the venous and arterial cannula for cardiopulmonary bypass (CPB), which is inserted in the respective femoral vein or artery and navigated to the right atrium or descending aorta under TEE guidance [37]. In the case of the endo-aortic balloon occlusion technique, as opposed to direct transthoracic aortic clamping, TEE provides safe and reliable guidance of the balloon for optimal placement [38,39]. After valve implantation, TEE is used to check valve patency and to identify procedure-associated complications [37,40].

TEE usage varies among the studies: Kubitz et al., Petersen et al., and Gebauer et al. used it to monitor all patients [12,13,15]. Zaouter et al. reserved using TEE for ERAS patients mainly to complement and monitor the intraoperative goal-directed therapy (GDT), illustrating their targeted use within their protocol [11]. Ertugay et al. utilized TEE intraoperatively to confirm the success of valve surgery and guide anesthetic management [17]. Pitts et al. further used TEE to guide the placement of the endo-aortic balloon occlusion [18]. Berretta et al. and Stock et al. did not provide specific details regarding their use of TEE [14,16]. Especially as a component of an early postoperative GDT concept, intraoperative TEE may guide perioperative resuscitation without the use of a pulmonary artery catheter (see Section 3.4.4).

#### 3.4.2. Protective Lung Ventilation

Lung-protective ventilation is recommended by the ERAS consensus statement for cardiac surgery [10]. This strategy typically involves using lower tidal volumes during ventilation (6–8 mL/kg predicted body weight), maintaining the driving pressure under 16 cm H_2_O, applying positive end-expiratory pressure (PEEP) of 5 cm H_2_O or higher, and using alveolar recruitment maneuvers, which have been shown to significantly reduce the risk of ventilation-associated complications after surgery and lead to improved outcomes for patients [41]. Berretta et al. described a protective lung ventilation protocol achieved by an alveolar recruitment using a temporary PEEP of 10 cm H_2_O, while Zaouter et al. and Ertugay et al. ventilated with small tidal volumes during CPB (see Section 3.4.3) to prevent alveolar collapse, atelectasis, and hypoxemia [11,14,17]. As the protocols prioritized on-table extubation and minimizing mechanical ventilation, most did not present a specific protective lung ventilation protocol [12,13,15,16,17,18].

#### 3.4.3. Ventilation on Cardiopulmonary Bypass

CPB typically allows for the cessation of lung ventilation to improve surgical exposure, but observational data supports using positive pressure during cardiac surgery [10]. Zaouter et al. maintained ventilation during CPB with a tidal volume of 3 mL/kg ideal body weight, a PEEP of 5 cm H_2_O, and a fraction of inspired oxygen of 0.35 [11]. Ertugay et al. used tidal volumes of 4–6 mL/kg and a PEEP of 5–10 cm H_2_O [17]. However, the protective benefits of these strategies and their impact on ERAS outcomes remain uncertain [10].

For RALT, ventilation during CPB can be performed using a single-lumen endotracheal tube, facilitating fast-track anesthesia. Effective coordination between the surgeon and anesthesiologist is essential to manage intermittent lung deflation and ensure adequate exposure during CPB [42].

#### 3.4.4. Use of Pulmonary Artery Catheters

None of the ERAS programs recommend the routine use of pulmonary artery catheters [11,12,13,14,15,16,17,18], with one study explicitly excluding their use [11]. They are not advised, as they do not decrease morbidity and mortality in a low-risk population while leading to a longer LOS and higher costs [10]. Especially in terms of GDT (see Section 3.6.1), they may not provide additional benefits in the MIVS ERAS patient group.

#### 3.4.5. Central Nervous System Monitoring

Central nervous system monitoring may help in the detection of cerebral hypoperfusion, but it is not necessarily a standard part of ERAS pathways. Clinical studies are still inconclusive, especially since classical ERAS parameters have not yet shown reproducible improvement through neurological monitoring [10]. Nonetheless, Kubitz et al., Ertugay et al., and Pitts et al. used near-infrared spectroscopy for cerebral perfusion and bispectral index monitoring to reduce the risk of cognitive decline [12,17,18].

#### 3.4.6. Postoperative Nausea and Vomiting Prevention

Following ERAS recommendations, PONV prevention should be approached multimodally [10]. Recommended practices include propofol for induction and maintenance of anesthesia, 4–8 mg of intravenous dexamethasone at induction, and 4 mg of intravenous ondansetron at the end of surgery [43]. If multiple risk factors are present or initial prophylaxis fails, 0.625 mg intravenous droperidol may be added [43]. PONV protocols from Kubitz et al., Stock et al., Ertugay et al., and Pitts et al. shared dexamethasone and ondansetron as core elements, respectively, with unique adjustments: the addition of droperidol [12], the addition of dimenhydrinate [16], adherence to the core approach [17], or preoperative benzodiazepine avoidance [18]. Zaouter et al. only used dexamethasone regularly [11], while Berretta et al. did not mention PONV as an ERAS component [14].

#### 3.4.7. Goal-Directed Perfusion

The ERAS consensus statement highlights the oxygen delivery index (DO_2_I) as the primary target for goal-directed perfusion (GDP), marking a paradigm shift from traditional body surface area-adjusted flow rates to physiologically guided targets [10,44]. A DO_2_I of ≥280 mL/min/m^2^ is recognized as the critical threshold to prevent oxygen and metabolic insufficiency and reduce end-organ dysfunction during CPB at moderate hypothermia (32–34 °C) [45,46].

Pitts et al. implemented GDP in line with these recommendations in their MIVS cohort by using a DO_2_I of ≥280 mL/min/m^2^ at 34 °C mild hypothermia [18]. By contrast, the studies by Kubitz et al. and Petersen et al. were conducted before this shift and followed then-standard body surface area-adjusted flow rates of ≥3.0 L/min/m^2^ [47]. They maintained CPB flow above 3.2 L/min/m^2^ and cooled to 32–33 °C, rewarming to 37 °C at the end of the procedure [12,13]. Based on TEE findings, Kubitz et al. adjusted noradrenaline to maintain a mean arterial pressure above 60 mmHg and prevent NIRS decreases greater than 10%, adding epinephrine during weaning from CPB [12]. Ertugay et al. have recently begun exploring GDP systems without presenting their concept yet [17].

### 3.5. Postoperative Process Measures

Postoperative elements are presented in Table 2, rows 15–18.

#### 3.5.1. Early Extubation Strategies

Early extubation has been a key component of previous fast-track programs. It has been integrated into most MIVS ERAS protocols, where on-table extubation is widely used whenever feasible (see Section 3.5.2) [12,13,14,15,16,17].

Noninvasive ventilation strategies were implemented by Zaouter et al., Kubitz et al., and Petersen et al. to support early extubation and reduce pulmonary complications [11,12,13]. To support early extubation, restrictive but carefully administered fluids, Minimally Invasive Extracorporeal Circulation (MiECC), adequate blood pressure control, and pain management are of utmost importance [48]. Additionally, systematic preoperative screening is important to detect pulmonary conditions such as severe COPD, which may carry a relevant risk for fast-track extubation [49]. Early extubation should be abandoned in cases of myocardial failure, hemodynamic instability, respiratory failure, or hypoxia, as well as mediastinal bleeding [48].

#### 3.5.2. Intraoperative Extubation

Most studies aimed for on-table extubation [11,12,13,14,15,16], while Ertugay et al. aim to achieve it as their next ERAS milestone [17]. Zaouter et al. successfully achieved on-table extubation in 13 of 23 ERAS patients, while time to extubation was not significantly reduced (*p* = 0.083). By contrast, Pitts et al. used a post-anesthesia care unit (PACU)-based strategy, achieving extubation after 140 (100–173) minutes in the ERAS group vs. 219 (153–333) minutes in the control group (*p* = 0.018), extubating after core temperature reached ≥36 °C, and absence of muscle relaxation or relevant pathologies under balanced metabolic measures were confirmed [18].

Retrospective data showed that extubation in the operating room is associated with reduced financial costs after cardiac surgery [50]. However, it might not be feasible for every department and may depend on local personnel, structural and logistical resources, considering that operating room costs make up more than half of the total patient costs [13]. Agitation and drowsiness, typically seen as reasons for delayed extubation, were not found to hinder on-table extubation as explored by Stock et al. [16].

#### 3.5.3. Acute Kidney Injury Prevention and Management

Most studies examined did not implement specific measures to prevent acute kidney injury (AKI). Usually, diuresis stimulation was either initiated by fluids or furosemide. Loop diuretics are frequently used after cardiac surgery to treat AKI induced by surgery and concomitant oliguria, but their role in AKI prevention remains uncertain [51,52]. It is recommended that measures be based on the KDIGO guidelines, especially in higher-risk patients. This includes avoiding nephrotoxic drugs for 48 h (e.g., AT II blockers, ACE inhibitors), avoiding hyperglycemia, monitoring serum creatinine levels and urine output monitoring, and maintaining fluid balance [10,53]. Goal-directed perfusion with a DO_2_I threshold of ≥280 mL/min/m^2^ may further reduce stage 1 AKI in cardiac surgery patients (see Section 3.4.7) [45]. Accordingly, Pitts et al. used this GDP strategy (DO_2_I ≥ 280 mL/min/m^2^, mild hypothermia 34 °C) [18]. Kubitz et al. included moderate hypothermia during CPB [12], while Berretta et al. used kidney biomarkers for early detection of AKI and therapy initiation in both groups equally [14], which highlights the diverging approaches in this topic [10].

#### 3.5.4. Postoperative Activity and Sternal Precautions

Early physiotherapy has been an integral part of ERAS programs and is associated with shorter ICU stays [54]. Berretta et al. attribute the reduced incidence of respiratory insufficiency [ERAS: 1 (0.7%) vs. control: 5 (3.3%); *p* = 0.04] to the immediate rehabilitation by a dedicated respiratory therapist team hours after surgery [14]. While Petersen et al. calculated a doubling of physiotherapy costs through their ERAS protocol (ERAS: EUR 188.8 ± 78.7 vs. control: EUR 94.5 ± 92.9; *p* < 0.001), total in-hospital costs were reduced by EUR 1087.2 per patient (*p* = 0.003), outweighing these additional expenses by a factor of 11.5 [13].

During mobilization, patients with sternotomy need to stabilize the sternum with counter pressure placed anteriorly [55]. However, this is not entirely applicable if full median sternotomy is avoided and a RALT, trans-axillary, or partial j-hemisternotomy approach is used. Here, MIVS reduces morbidity and represents an integral part of an ERAS approach, allowing patients to recover and regain mobility faster [56]. Physiotherapy, including respiratory therapy, should be adapted according to each patient’s individual needs and start on the day of surgery—if no contraindications are present. All studies analyzed focused on extensive mobilization through ERAS protocols and began on the day of surgery until discharge [11,12,13,14,15,16,17,18]. Non-ERAS patients, by contrast, appear to begin mobilization after 24 h or as late as the third postoperative day [11,14].

Standard physiotherapy protocols usually begin with breathing exercises and escalate over five days to reintroduce full mobilization [57]. ERAS mobilization progresses quicker, including ergometer exercising and stair climbing on the second to third day after surgery [12]. It should be considered that early mobilization synergistically benefits from other key ERAS elements, such as early removal of chest tubes (see Section 3.7.2), minimization of PONV (see Section 3.4.6) and adequate hemodynamics, e.g., supported by GDT (see Section 3.6.1). Early mobilization, especially getting out of bed on the day of surgery into a standing position, is a common hurdle in recovery and requires professional assistance from physiotherapists [54].

### 3.6. Multiphase Process Measures

Multiphase elements are presented in Table 2, rows 19–25.

#### 3.6.1. Goal-Directed Therapy

GDT refers to a systematic approach that aims to reach specific hemodynamic targets by administering fluids, vasopressors, and inotropic medications. A key principle of GDT is the identification of patients who respond to fluid administration using dynamic assessments such as stroke volume, stroke volume responsiveness, and pulse pressure variation. This enables appropriate resuscitation measures, an important component in ERAS programs. All studies included some form of GDT, which may reduce complications, ICU, and total hospital LOS [58,59]. However, GDT algorithms are not standardized and differ between every center. Zaouter et al. used their GDT algorithm, which starts with a fluid challenge and potentially escalates to catecholamine use when blood pressure remains below 20% of baseline [11]. Pitts et al. relied on an experienced ERAS anesthesiologist to perform GDT, incorporating continuous evaluations of radial arterial pressure, stroke volume, and echocardiography for precise hemodynamic management [18]. Kubitz et al. aimed for a strict neutral to negative fluid balance goal after 24 h. This approach was complemented by using hemofiltration during CPB (see Section 3.9.1) to extract the priming volume; however, this approach did not receive further interest [12,13,15,19]. Berretta et al. followed a basic GDT approach using fluids, vasopressors, and inotropes to prevent hypotension and low cardiac output, which was equally applied to both groups, though no detailed protocol was described [14]. Ertugay et al. also emphasized goal-directed fluid therapy in the ICU phase, focusing on vasopressor and fluid optimization without detailing a specific protocol [17]. Finally, albumin may be considered in patients requiring additional crystalloid resuscitation to avoid excessive positive fluid balance. In contrast, hydroxyethyl starch is not recommended for routine use due to its potential association with increased bleeding risks [60,61,62,63,64].

#### 3.6.2. Opioid-Sparing Pain Management

While opioids have historically been central to cardiac surgery analgesia, their association with prolonged hospital stays and adverse effects has prompted modern ERAS protocols to prioritize minimizing their use and adopting multimodal pain management strategies [65]. While mitral MIVS is already associated with lower pain scores (MD −1.06; 95% CI: −3.96 to 0.75), opioid use becomes considerably dispensable after chest tube removal [66,67]. The ERAS Cardiac Working Group introduced a Turnkey Order Set to address the current variability of opioid use, offering clinicians a standardized multimodal pain management framework. Their approach integrates a range of agents to optimize analgesia while minimizing opioid reliance [68]. Patient education and psychosocial support further complement pharmacologic measures, enhancing recovery and reducing opioid use [65,68]. However, nonsteroidal anti-inflammatory drug use requires caution due to its potential link to thrombotic cardiovascular complications [65,68].

The analyzed studies highlight diverse multimodal pain management approaches, balancing shared foundational elements with institution-specific adaptations. Zaouter et al. implemented an advanced multimodal analgesia protocol using up to eight agents, with acetaminophen, nefopam, magnesium, and pregabalin applied in over 90% of ERAS patients. This approach significantly reduced opioid consumption compared to controls [11]. Stock, Kubitz, and Pitts shared a similar same-day analgesia strategy, prioritizing piritramide and metamizole immediately postoperatively [12,16,18]. Stock and Pitts both introduced structured tapering protocols, discontinuing opioids by the third to fifth postoperative day, and emphasized cessation following chest drain removal [16,18]. Stock further incorporated scheduled metamizole, oxycodone/naloxone, and pregabalin (for lateral thoracotomy) starting on the first postoperative day, with early chest drain removal on the day of surgery (see Section 3.7.2) to minimize analgesic requirements [16]. Kubitz and Pitts focused on regular non-opioid analgesics such as metamizole and paracetamol to support opioid reduction [12,18]. Ertugay employed a multimodal regimen centered on paracetamol, dextromethorphan, and tramadol, reflecting a tailored approach to postoperative analgesia [17].

#### 3.6.3. Regional Analgesia

Infiltrative techniques and intercostal nerve blocks have been widely utilized. Nevertheless, the benefits of wound infiltration have not consistently outperformed its omission, leading to a shift toward regional nerve blocks in modern guidelines [65]. Most studies in this systematic review employed regional analgesia, ranging from wound infiltration with sodium channel blockers like ropivacaine to catheter-based local anesthesia [11,12,13,14,15,17]. Stock and Pitts highlighted using ultrasound-guided regional nerve blocks, such as serratus or parasternal blocks, depending on the surgical access route to reduce intraoperative and postoperative analgesic requirements [16,18]. These blocks, which are particularly effective in ERAS protocols aiming for fast recovery after MIVS, are quick, safe, and optimally performed under sterile conditions at the end of surgery. Emerging methods like cryotherapy and local perfusion catheters further reflect the future adaptability of modern ERAS protocols [17]. The growing prevalence of regional nerve blocks underscores their role as a cornerstone of multimodal pain management in ERAS strategies [65].

#### 3.6.4. Patient Blood Management Program

Postoperative transfusion triggers between a hemoglobin level of 7 and 8 g/dL seem safe, while accounting for patient symptoms and clinical presentation [60,69]. Four studies used such triggers between a hemoglobin of 7.2 g/dL [11] and 7.5 g/dL [12,13,15], which did not affect transfusion rates in such small cohorts. Pitts and Berretta utilized MiECC with autologous retrograde priming to reduce hemodilution [14,18,70,71]. Ertugay’s approach, in contrast, incorporated normovolemic hemodilution and antifibrinolytics but still exhibited a relatively high transfusion rate, likely reflecting their learning curve [17].

Future studies might research the implementation of recombinant human erythropoietin. Though currently it is not routinely recommended, as relevant Hb increases might take weeks and come with serious health risks [69].

#### 3.6.5. Postoperative Atrial Fibrillation Prevention

Prophylaxis for atrial fibrillation prevention is an effective tool to prevent event rates and may reduce the total length of hospital stay and financial cost [72].

Beta-blockers are recommended to be continued throughout the perioperative period. They are the first-line medication for the treatment of atrial fibrillation, although none of the included studies explicitly addressed their continuation [72,73].

Prophylactic use of amiodarone has been shown to be effective in reducing postoperative atrial fibrillation events, but acute and long-term complications must be considered [10,73]. Kubitz et al. implemented a 24 h continuous amiodarone infusion for patients with risk factors (enlarged left atrium, reduced LVEF, and history of atrial fibrillation) [12]. Similarly, Petersen et al. described a low-dose amiodarone prophylaxis [13].

Atrial fibrillation might also be reduced through the use of MiECC (see Section 3.8.2) or a posterior pericardiotomy (see Section 3.8.3), both of which reduce the inflammatory response [70,74,75].

The role of potassium supplementation in cardiac surgery has recently been minimized, as achieving high-normal potassium is not associated with lowering dysrhythmias or adverse clinical events [76].

The latest STS guidelines recommend concomitant surgical ablation for atrial fibrillation during nonemergent mitral valve surgery and other cardiac surgeries, as well as left atrial appendage closure to reduce thromboembolic risk [77]. The ablation can be achieved by RALT access and was performed in roughly two-thirds of atrial fibrillation patients, as described by four of the included studies prior to the publication of these guidelines [12,15,17,18]. Factors such as left atrial size, advanced age, and incomplete ablation may impact rhythm conversion rates, highlighting the importance of patient selection and precise execution. Optimal perioperative antiarrhythmic and anticoagulation strategies require further study, while patients often receive amiodarone for 2–3 months [77].

#### 3.6.6. Systematic Delirium Screening and Prevention

Delirium screening can easily be implemented in most hospitals and should be conducted once per nursing shift on the first day after surgery. Suitable screening tools include the Confusion Assessment Method for the Intensive Care Unit and the Intensive Care Unit Delirium Screening Checklist [10], which are applied irregularly across all included studies. Delirium after ERAS MIVS occurred in up to 6% of patients [12,14,15,18], notably lower than the population incidence of up to 50% [78]. This discrepancy may reflect reduced mechanical ventilation times, ICU stays, selection of younger, healthier patients with lower NYHA classes and higher LVEF [78], and a limited focus on detecting delirium. Only one included study was associated with significantly reduced delirium rates, attributing this to the early removal of restraining lines (e.g., catheters and drains) and early physiotherapy; however, it did not employ a standardized delirium screening regimen [14]. Current literature recommends abstaining from benzodiazepines for older patients, who are typically not included in ERAS programs [30]. Thus, one study explicitly excluded the use of benzodiazepines as premedication [11], while others included it in the ERAS protocol [12,14]. Berretta and Pitts emphasized early family contact after surgery, which can help reorient patients and is a core delirium prevention technique [10,14,18]. Ertugay et al. also emphasized preoperative alcohol cessation as a potential preventive measure [17]. Therefore, ERAS protocols should integrate a regular delirium screening to identify and treat postoperative delirium early and effectively [10].

#### 3.6.7. Surgical Site Infection Prevention Bundle

Multiple factors influence the likelihood of developing postoperative surgical site infections (SSI), some of which can be prevented, but not all. Fixed determinants such as surgical risk index, high body mass index, and diabetes pose the greatest risks and are further exacerbated by heart failure, renal dysfunction, smoking, COPD, or MRSA [10,79,80]. These factors can be identified through preoperative screening measures, with two studies including smoking cessation as part of their SSI bundle [11,17]. Modifiable preoperative factors such as diabetic control, treatment of preexisting infections, and optimization of the patient’s nutritional profile are also crucial [10,79].

Intraoperative measures can be improved through excellent hemostasis, meticulous surgical techniques, and avoidance of bone wax in j-hemisternotomy [10,79]. Postoperatively, rapid extubation, early removal of invasive catheters, continuation of antibiotic prophylaxis according to local recommendations, and adherence to restrictive transfusion triggers are recommended [10,79]. The foundational elements of SSI prevention, such as antibiotic prophylaxis and glycemic control, were emphasized by Ertugay, Berretta, and Pitts [14,17,18]. Additionally, Kubitz et al. emphasized maintaining postoperative normothermia [12], while Berretta et al. included smoking cessation [14].

### 3.7. Process Measures Not Graded by ERAS Guidelines

#### 3.7.1. Minimally Invasive Surgery Approach

MIVS facilitates ERAS protocols in several ways and carries great synergism to reduce morbidity. It is associated with less trauma, reduced opioid consumption, and the required number of chest tubes—ideally only one [81]. Additionally, it promotes rigorous physiotherapy by preserving the stabilizing sternal integrity [56].

Minimally invasive mitral surgery is performed in a supine position with a pillow under the right scapula to elevate the right thorax [81]. The patient’s arm is positioned at the patient’s side with the shoulder dropped down, exposing the thorax for a lateral thoracotomy through the fourth intercostal space [81]. The skin incision is made in the submammary crease in women and inframammary in men [81]. Alternatively, a periareolar incision can be used in male patients, which has been demonstrated to be safe while also being cosmetically more appealing [82]. Large meta-analyses suggest that mitral MIVS reduces LOS by 2 days compared to sternotomy and may be even more beneficial in redo mitral surgery [67,83,84,85].

For aortic valve surgery, a supine position should also be taken as well. If performed through the RALT or trans-axillary approach, the arm should be positioned over the patient’s head [86]. For lateral thoracotomy, the positioning is identical to mitral lateral thoracotomy, with the arm to the side and a pillow under the right scapula [86]. In contrast, j-hemisternotomy uses a standard supine position. A Cochrane review indicates that aortic MIVS may reduce LOS by about 1 day, but evidence remains uncertain due to heterogeneity [3].

Robotic surgical techniques are progressing as a further option in MIVS. In one study, Yost et al. achieved lower morbidity and equal mortality and readmission rates in the 34% of patients (57/169) discharged on the first or second postoperative day. Of these, 70% were MIVS. An ERAS protocol was not implemented, showcasing the advantages of MIVS [87]. A meta-analysis by Williams et al. shows robotic mitral MIVS shortens LOS by up to 2 days, though the data also revealed considerable heterogeneity [88].

#### 3.7.2. Removal of Chest Tubes, Catheters, and Pacemaker Wires

Early removal of chest tubes, catheters, and pacemaker wires is crucial for reducing postoperative complications such as delirium and improving patient mobility [89]. Various approaches were therefore included in the included ERAS protocols. It can be associated with barriers to clinical practice if healthcare professionals are not educated about the ERAS program. This highlights the advantages of a multidisciplinary team (see Section 3.2.2) involving all disciplines in the perioperative patient pathway [26].

Early chest tube removal is integral to all included ERAS protocols [11,12,13,14,15,16,17,18]. Recent data support an aggressive removal of chest tubes on the first postoperative day after MIVS, which does not compromise patient safety [90]. The most ambitious protocol was Stock et al., where chest tubes were removed on the day of surgery if the output remained <500 mL in the first 8 h and no air leak was present [16], an approach requiring further validation. Diagnostic tools like pleural sonography and chest X-rays should be used liberally after removal, and fluid overload should be avoided, especially in patients with preoperative renal insufficiency or reduced left ventricular ejection fraction.

It appears to be safe to remove central line access by 12 to 24 h after surgery [11,12,17]. Early removal is preferred to reduce central line-associated bloodstream infections as part of an SSI prevention bundle (see Section 3.6.7). In arrhythmias requiring intravenous amiodarone administration, the central catheter may be removed later [79]. Arterial lines should be removed once stable hemodynamics and adequate gas exchange are achieved without catecholamines under nasal cannula oxygen.

Early removal of the pacemaker wire may be the last obstacle to the patient’s mobilization. Hemodynamic stability, freedom from arrhythmia, and absence of pacemaker dependency over the last 24 h should be ensured and confirmed by ECG monitoring [91]. The administration of amiodarone and beta-blockers to control new-onset postoperative atrial fibrillation should be awaited before the pacemaker wires are removed, as AV blockages may occur. The wires can safely be removed once a stable sinus rhythm is established [92]. Although there is a slightly higher risk of arrhythmia with earlier removal, waiting until the third postoperative day, as described by Kubitz, may be unnecessary, and waiting even longer, such as 4 days, prolongs the hospital stay [12,91]. In patients with preoperative right bundle branch block, pacemaker wires should be removed later due to a higher risk of complete AV block [93]. Pitts et al. have recently tested omitting epicardial pacing wires in patients with hemodynamically stable sinus rhythm at the end of surgery [18], though this approach may not suit all cases.

A comprehensive comparison of the ERAS elements implemented in the included studies, based on the recommendations of the consensus statement, is presented in Table 3. This table highlights institutional variations in ERAS application across different centers.

### 3.8. Promising Process Measures Not Included in ERAS Guidelines

While not currently part of ERAS cardiac recommendations, the following measures can potentially advance future ERAS protocols in MIVS patients.

#### 3.8.1. Shortening ICU Stay

The studies by Stock et al. and Pitts et al. explored strategies to reduce ICU stays in MIVS ERAS protocols through same-day discharge from a PACU to the general ward [16,18]. This concept was first introduced by Haanschoten et al. in a cohort of 5367 patients undergoing non-complex cardiac surgery. Of these, 84% (*n* = 4510) were successfully discharged from the PACU on the same day [94]. Stock et al. assessed the feasibility of transferring patients to the general ward after 6 h in the PACU. Of 358 eligible ERAS patients, 297 met the inclusion criteria of uneventful surgery, on-table extubation, and cardiopulmonary stability. A total of 90% of these patients (*n* = 266) met further safety criteria for it to be feasible for them to be discharged to the general ward after 6 h. Safety criteria were mainly missed due to continued vasopressor need [16]. In contrast, Pitts et al. implemented a PACU protocol targeting evening transfer for all 45 ERAS patients. Their protocol was successful in 80% of cases, with failures primarily due to bleeding revision (*n* = 4) or respiratory complications (*n* = 4) [18].

All three studies emphasize strict eligibility criteria for general ward discharge. These include unremarkable imaging of the pericardial and pleural spaces (via x-ray and ultrasound) and chest tube drainage < 50 mL/h. Patients also needed to be alert and oriented, free of catecholamines, maintain adequate oxygen saturation with <5 L/min flow of oxygen via nasal cannula, and achieve sufficient analgesia [16,18,94].

Petersen et al. reported a significant reduction in ICU costs associated with a shorter ICU stay after MIVS in the ERAS vs. control group (26.5 ± 25.2 h vs. 46.6 ± 44.9 h; *p* = 0.010). This ICU reduction resulted in cost savings of EUR 925.7 per patient (EUR 1431.9 ± EUR 1369.2 vs. EUR 2294.9 ± EUR 2185.5; *p* = 0.007). These findings highlight the economic benefits of shorter ICU stays [13], which could be amplified by implementing a Day 0 concept.

#### 3.8.2. Minimally Invasive Extracorporeal Circulation

MiECC extends the minimally invasive surgical approach to the CPB by integrating the latest advances into a closed-loop circuit [71]. This approach reduces hemodilution, leading to fewer red blood cell transfusions, less atrial fibrillation, preserved renal function, and better myocardial protection [71]. MiECC was first utilized in bypass surgery, where it successfully promoted ERAS pathways and is now being integrated into MIVS [14,71]. The benefits of MiECC are achieved through membrane oxygenators, centrifugal pumps, biocompatible and miniature circuits, and avoidance of cardiotomy suction if possible [71]. MiECC has shown a 25% relative risk reduction in serious complications and serious adverse events after cardiac surgery [74], highlighting its significance in improving patient outcomes. Additionally, it frees the surgical field for the surgeon, facilitating a smoother operation [70,71].

Pitts et al. applied MiECC in all ERAS patients [18], and Berretta et al. used it in approximately one-third of their ERAS group and one-sixth of their control group [14]. In contrast, Zaouter et al. applied it in two-thirds of both patient groups [11]. To further enhance MiECC, percutaneous cannulation of the femoral vessels may be an additional safe and uncomplicated tool, avoiding open surgical cut down to the femoral vessels and groin complications [95]. Retrograde priming can further reduce hemodilution, which may reduce perioperative transfusions [96].

#### 3.8.3. Posterior Pericardiotomy

Posterior left pericardiotomy, a technique that remains underrepresented, is associated with a significant decrease in atrial fibrillation, early and late pericardial effusion, and cardiac tamponade, ultimately shortening the length of hospital stay [75,97]. While pleural effusions are slightly increased, it does not lead to a higher rate of pulmonary complications [97]. However, the viability of this technique in MIVS may only account for j-hemisternotomy. Non-sternotomy approaches may not offer surgical exposure to perform posterior left pericardiotomy.

#### 3.8.4. Interpersonal Advancements

The psychological burden of cardiac surgery is often overlooked in ERAS protocols, as evidenced by Schmidt et al.’s interviews with MIVS patients undergoing ERAS programs [21]. Their study underscores the importance of a dedicated ERAS nurse’s care, meeting all staff before surgery, and direct transfer to rehabilitation from the hospital stay [21]. Surgeons can further improve patient care through implementing shared decision making principles by visiting patients before and after surgery [21,98]. The ongoing INCREASE trial incorporates these findings, highlighting the implementation of a dedicated ERAS nurse as a key factor in improving patient outcomes, which is a novel approach in MIVS trials [19]. Several studies addressed psychological support by including a psychologist in the ERAS team [12,13,15,16,17]. An ERAS coordinator or nurse champion must guide these professions to ensure effective interdisciplinary collaboration and optimal patient care [26].

#### 3.8.5. Anticoagulation After MIVS

Since 2021, the European guidelines for the management of valvular heart disease have supported the use of direct oral anticoagulants (DOACs) or aspirin over vitamin K antagonists (VKAs) after biological heart valve surgery in specific contexts [5]. For aortic biological valve implantation, DOACs are recommended, while for mitral biological valves, DOACs may be considered in the presence of atrial fibrillation [5]. When no atrial fibrillation or other anticoagulation indications are present, low-dose aspirin is recommended for biological aortic valves [5]. The 2024 guidelines on perioperative medication in adult cardiac surgery recommend low-dose aspirin for three months following mitral valve repairs without other anticoagulation indications. For mitral valve replacements without other anticoagulation indications, VKAs are advised for three months, followed by low-dose aspirin [73].

While specific anticoagulation protocols were not consistently detailed in the included studies, VKAs were likely the standard for the first three months after mitral or tricuspid valve surgery, as recommended by the 2017 guidelines [99]. The use of VKAs requires careful titration and regular monitoring, which likely contributed to longer LOS under these older guidelines. By contrast, the updated 2024 guidelines highlight low-dose aspirin and DOACs as viable alternatives in specific contexts, offering simplified anticoagulation management and reducing the need for monitoring. DOACs can be initiated as early as the third postoperative day, following pacemaker wire removal and confirmation of no pericardial effusion, offering potential advantages in postoperative recovery and reintegration into daily life [5,91].

As previously discussed, the optimal anticoagulation strategy following atrial ablation and left atrial appendage closure remains a topic of ongoing debate, with no definitive consensus established to date (see Section 3.6.5) [77].

#### 3.8.6. Nutritional Intake

Non-ERAS patients sometimes begin oral feeding after 24 to 72 h post-surgery [11,14]. Recent research suggests cardiac surgery patients receive inadequate nutrition support after surgery and can be offered food much earlier, although there are still no widely accepted protocols for non-intensive care patients [100,101]. The ERAS protocol with the earliest restarting of food intake is Kubitz et al., where oral nutrition is restarted 6 h after surgery [12]. Therefore, early nutritional intake and achieving nutritional targets (e.g., energy and protein) is crucial in ERAS protocols, as it can enhance recovery and improve patient outcomes.

### 3.9. Non-Promising Process Measures Not Included in ERAS Guidelines

#### 3.9.1. Hemofiltration

In the trial by Kubitz et al., intraoperative hemofiltration was used during CPB to manage fluid balance by removing priming volume, aligning with their goal of achieving a neutral to negative fluid balance 24 h post-surgery [12]. However, this practice was not continued in their subsequent INCREASE trial [19]. Hemofiltration adds complexity and has been linked to increased lactate levels and inotropic use, suggesting it should be reserved for specific cases rather than being a standard component of ERAS protocols [102]. The balance of benefits and risks, particularly regarding the impact on renal function, requires careful consideration, leading to the non-general use of this technique. Alternatives, such as retrograde priming to reduce priming volume (see Section 3.6.4), should be considered instead.

## 4. Discussion

Several studies have reviewed ERAS programs in cardiac surgery, highlighting significant interest in this topic, whereas limited evidence exists about the clinical significance in MIVS patients. Maj et al. examined ERAS protocols in minimally invasive cardiac surgery procedures, while Malvindi et al. recently focused on ERAS in cardiac valve surgeries [9,103]. These studies suggest significant heterogeneity in ERAS protocols across different centers. Existing evidence is derived mainly from smaller studies, including three pilot trials [12,16,17], which limits generalizability and highlights the necessity for further research to strengthen the evidence base. Together, these studies included a total of 1287 patients, with 842 following ERAS protocols and 445 serving as controls. Research suggests that MIVS alone may not lead to benefits such as a reduction in LOS unless coupled with an ERAS protocol to maximize patient benefit [3,9,104]. As elective MIVS is safer than ever, focusing on secondary outcomes, such as reducing complications and safely shortening LOS, becomes essential to improving patients’ quality of life while saving costs through ERAS [14].

Current MIVS ERAS protocols implemented between 9 and 18 of 24 different measures recommended by the recent ERAS cardiac consensus guideline (Table 3) [10]. Some straightforward yet potentially impactful measures are often omitted; for example, only Berretta et al. and Pitts et al. incorporated early family contact; likewise, only Zaouter et al. included a dedicated protective lung ventilation protocol, while none included structured delirium screenings [11,14,18]. A lack of detailed reporting of the ERAS protocols often obscures why specific measures were not implemented. A critical improvement would be to broaden the existing ERAS protocols by integrating a wider range of these measures.

Indeed, the implementation process may represent the Achilles heel and is mainly influenced by limited resources and insufficient buy-in from the different healthcare disciplines. Here, maximum transparency of the ERAS protocols seems mandatory to gain commitment and support from every discipline included in the perioperative patient pathway. Ideally, this is achieved by an MDT under the guidance of an ERAS coordinator [25,26].

Shortening the ICU and total LOS is an essential aspect of ERAS protocols. MIVS ERAS studies showed ICU LOS reductions of 4–20 h or even bypassing ICU entirely, while the total length of stay was likewise reduced by at least one day without increasing complications or compromising patients’ safety [11,13,14,15,18]. Same-day PACU-to-ward approaches, tested for feasibility by Stock et al. (74% meeting all safety criteria) and successfully implemented by Pitts et al. (80% success rate), highlight this potential to reduce the ICU dependency without compromising safety [16,18]. These reductions through the implementation of ERAS protocols contribute to improved patient recovery and significant cost savings. Cost analyses, such as those by Petersen et al., highlight the economic impact of ICU reductions, the largest contributor to savings. ICU time decreased from 46.6 ± 44.9 h in the control group to 26.5 ± 25.2 h in the ERAS group, resulting in EUR 925 saved per patient (*p* = 0.007) [10]. Through other reductions in operating room, general ward stay, and internal activity allocations, total savings amounted to EUR 1909.8 per patient (*p* = 0.006) [13].

While these data show the feasibility and safety of same-day approaches, they require institutional resources and strict patient selection to mitigate risks [16]. Pitts et al. conducted a mandatory MDT visit and regular safety checkpoints likewise suggested by Stock et al. [16,18]: at transfer, patients had to be alert and oriented, be hemodynamically stable without catecholamines, receive <5 L O_2_ and have adequate pain control, unremarkable x-ray, ultrasound, and drain volumes (<50 mL/h) [16,18]. Other centers have successfully implemented early discharge protocols in MIVS where patients are discharged to home on the second to third postoperative day, likewise achieving significant cost savings while maintaining safety [105]. To enable these shorter LOS, novel approaches of removing drains 8 h after surgery or omitting epicardial pacemaker wires have been tested [16,18], yet they lack data from bigger trials. While major complications likely occur independent of an omitted ICU stay [16], more extensive trials are also required to validate these strategies for ERAS in MIVS to assess their general applicability and distinguish MIVS ERAS from conventional valve surgery. Future studies should consider that such early discharge may shift costs to outpatient care, which should be accounted for in the overall cost analysis [19]. The wide variety of approaches emphasizes the importance of tailoring the ERAS protocol to the institutional capabilities and structural and personnel resources while respecting evidence-based measures.

Extending this to patient perception, Schmid et al. highlighted that patients did not particularly notice the changes under ERAS protocols due to a lack of understanding of slower, traditional recovery processes without them. Without reference points, healthcare providers can guide patients through early and rigorous measures. Effective patient education reinforces the benefits of ERAS protocols, ensuring adherence and promoting both physical and psychological well-being [21].

ERAS protocols provide patients with significantly higher levels of care compared to non-ERAS, offering significant benefits from early actions such as optimized nutrition and early physiotherapy. Conversely, patients in control groups often experience delayed initiation of these measures by modern cardiac surgery standards. Prominent examples are the first full meal on the fourth postoperative day or beginning physiotherapy very late, which limits their recovery [29,103]. These basic measures should be implemented less exclusively to ensure that all patients, including those in control groups, receive timely interventions. ERAS protocols should distinguish themselves by incorporating rigorous physiotherapy sessions tailored to patients’ needs, avoiding overly standardized and less intensive approaches.

ERAS programs, especially for MIVS, should be expanded and applied in more centers. Their components synergistically enhance overall recovery and reduce complications. However, there remains a need for more comprehensive data, such as reintegration into everyday life, 12-week, or 1-year outcomes compared to conventional approaches. With only one trial exploring ERAS in robotic MIVS, more research is needed to establish its role and potential benefits. Such data will be crucial to fully evaluate these programs’ safety and real-life benefits, guiding further refinement of ERAS protocols and subsequently the standard of care.

## 5. Limitations

This review is limited by the number of included studies, considering the limited availability of studies for ERAS in MIVS. The included studies were small, predominantly observational studies, limiting the generalizability and validity of their findings. Additionally, the wide variability and sometimes contradictory approaches of ERAS protocols complicate direct comparison and an overall conclusion. Variability in defining and diagnosing secondary outcomes like respiratory insufficiency, pneumonia, SSI, or delirium further limits comparability, underscoring the need for standardized reporting. Another limitation is that the current consensus statement was published after the ERAS protocols were developed [10]. This may have led to the omission of specific measures now considered standard or recommended. There is a lack of detailed reporting on the rationale behind specific measures included or excluded in the different studies, making comparison difficult. While acknowledging the sophistication of the studies, this review highlights the diverse approaches currently employed in MIVS ERAS programs. It underlines the need for more robust, large-scale research to validate and refine these protocols.

## 6. Conclusions

As ERAS programs become more prevalent in cardiac surgery, MIVS offers strong synergism in maximizing the benefits of these protocols. Across studies, ERAS protocols in MIVS consistently reduced ICU LOS by 4–20 h, with two protocols further demonstrating the safety of skipping ICU altogether. Total hospital LOS was shortened by at least one day, achieving significant cost savings while maintaining patient safety. These outcomes highlight the adaptability of ERAS protocols to institutional needs despite variability, with studies adopting between 9 and 18 of the 24 recommended measures.

Key elements such as on-table or early extubation, early mobilization, opioid-sparing pain management, and the timely removal of invasive devices were integral to these successes. Additionally, the use of minimally invasive approaches, including MiECC and endo-aortic clamping, further supported ERAS goals by reducing surgical trauma and accelerating recovery.

While the initial workload for implementation is considerable, these protocols lead to standardized hospital processes, greater efficiency, and a more patient-centered approach to care. The transition to widespread ERAS integration in more hospitals should be led by centers with expertise in MIVS and ERAS, serving as role models for others. Future studies should also evaluate long-term outcomes, including reintegration into daily life and quality of life, to further refine these protocols and expand their applicability. This universal application has the potential to significantly elevate the standard of care and maximize the positive impact of ERAS programs.

## Figures and Tables

**Table 1:** Overview of included studies.

Author	Short Description	Patient Characteristics (ERAS/Control)	Inclusion/Exclusion Criteria	Study Goals	Primary and Secondary Endpoints (ERAS vs. Control)
Zaouter et al.—2019 (Bordeaux, FRA) [11]	Observational cohort study Before and after trial Before: 09/2014–12/2014 After: 05/2015–11/2015 ERAS: *n* = 23 Control: *n* = 23	Age: 80 (74–82)/73 (68–82); *p* = 0.038 Sex (M/F): 14/9/16/7; *p* = 0.038 BMI: 26 (23–27)/28 (26–32); *p* = 0.022 CPB Time (min): 81 (75–85)/80 (73–90); *p* = 0.667	**Mini-sternotomy for aortic valve** Full/previous sternotomy Dementia Endocarditis Chronic RI	Extubation within 6 h of surgery (modified Reyes’ extubation criteria) Multidisciplinary Team agreed on evidence-based protocol	**Hospital LOS** (days): 7 (6.5–8) vs. 10 (9–13.5); *p* < 0.001 ICU-LOS (hours): 24 (24–28) vs. 28 (25–47); *p* = 0.003 ICU pain scores: 1.4 (0–2) vs. 2 (2–3); *p* = 0.035 Total post-op morphine: 2 (0–12) vs. 7 (3–12); *p* = 0.090
Kubitz et al.—2020 (Hamburg, GER) [12]	Pilot study Retrospective analysis of first ERAS patients 02/2017–07/2018 ERAS: *n* = 50 control: *n* = 0	Age: 51.9 ± 11.9/NA; *p* = NA Sex (M/F): 38/12/NA; *p* = NA BMI: 26.1 ± 3.1/NA; *p* = NA CPB Time (min): 137.8 ± 47.9/NA; *p* = NA	**MIC (mitral and aortic valve) incl. mini sternotomy** **Age < 70 years** Redo surgery Concomitant procedure requiring sternotomy Relevant comorbidities: i.e., prior stroke, terminal RI	On-table extubation, PACU to ICU, ward POD 1 Flow of >3.2 L/m^2^ BSA Multidisciplinary Team Assess ERAS feasibility and safety	**Safety and feasibility:** - in-hospital mortality: 0 (0%) - all patients considered fit for low/intermediate care unit 3.5 h after surgery ICU LOS (hours): 14.0 ± 7.4 Hospital LOS (days): 6.2 ± 2.9
Petersen et al.—2021 (Hamburg, GER) [13]	Observational cohort study Economic impact of ERACS in MIC ERAS and control simultaneously 02/2018–03/2019 ERAS: *n* = 61 Control: *n* = 69	Age: 50.7 ± 12.9/54.1 ± 9.5; *p* = 0.096 Sex (M/F): 47/14/52/17; *p* = 0.256 BMI: 26 (23–27)/28 (26–32); *p* = 0.022 CPB Time (min): 87 (73–108)/94 (77–112); *p* = 0.23	**MIC (mitral and aortic valve) incl. mini sternotomy** **Age < 70 years** Redo surgery Concomitant procedure requiring sternotomy Relevant comorbidities: i.e., frailty	On-table extubation, PACU to ICU, ward POD 1 Mix of costs per minute (e.g., OR time), selected costs, and total costs Multidisciplinary Team	**Difference in the average cost** (per patient in Euro EUR) total cost: 11,200.0 ± 3029.6 vs. 13,109.8 ± 4527.5; *p* = 0.006 - OR cost: 5518.4 ± 1.140.1 vs. 5990.9 ± 1495.5; *p* = 0.047 - ICU cost: 1431.9 ± 1369.2 vs. 2294.9 ± 2185.5; *p* = 0.007 - general ward cost: 1047.5 ± 504.6 vs. 1271.2 ± 596.3; *p* = 0.023 - physiotherapy: 188.8 ± 78.7 vs. 94.5 ± 92.9; *p* < 0.001 Cost savings (ca. 1000 Euro) ICU LOS (hours): 26.5 ± 25.2 vs. 46.6 ± 44.9; *p* = 0.010 Hospital LOS (days): 4.7 ± 2.2 vs. 5.6 ± 2.7, *p* not calculated
Beretta et al.—2023 (Ancona, ITA) [14]	Propensity-matched analysis 10/2016–07/2020 ERAS: *n* = 152 Control: *n* = 152 (after propensity matching)	Age: 69.6 ± 11.1/70 ± 11.9; *p* = 0.813 Sex (M/F): 78/74/84/68; *p* = 0.813 BMI: 26.2 ± 3.8/26.2 ± 4.5; *p* = NA CPB Time (min): 77 (64–96)/77 (63–101); *p* = NA	**Decision by Multidisciplinary Team** **MIC (mitral and aortic valves) incl. mini sternotomy** Urgency/emergency/previous/concomitant CS Severe hemodynamic instability Severe heart failure NYHA IV, LVEF < 30% and IABP Chronic RI/ dialysis or severe hepatic disease Severe chest wall deformities	Normothermic cardiopulmonary bypass management UFT-anesthesia with table extubation Immediate rehabilitation therapy and patient-family contact	**Mortality (at POD 30)**: 0 (0%) vs. 1 (0.7%); *p* = 0.9 **Neurologic adverse events** (at POD 30) - stroke: 0 (0%) vs. 2 (1.3%); *p* = 0.5 - delirium: 1 (0.7%) vs. 6 (3.9%); *p* = 0.04 ICU LOS (hours): 30 (24–52) vs. 40 (24–59); *p* = 0.03 Hospital LOS (days): 6 (5–7.7) vs. 7 (6–8); *p* = 0.04 Bleeding requiring re-exploration: 3 (2%) vs. 3 (2%) 1; *p* = 1 Resp. insufficiency: 1 (0.7) vs. 5 (3.3); *p* = 0.04 Estimated survival (at 12 months): 99.2% vs. 98.6%; *p* = 0.6
Gebauer et al.—2023 (Hamburg, GER) [15]	Observational cohort study Further developed ERAS protocol 02/2018–09/2020 ERAS: *n* = 101 Control: *n* = 111	Age: 56 ± 17/57.5 ± 13; *p* = 0.015 Sex (M/F): 74/27/79/32; *p* = 0.734 BMI: 25.7 ± 3.4/26.2 ± 3.3; *p* = 0.271 CPB Time (min): 130.5 ± 61/147 ± 81; *p* = 0.076	**MIC (mitral (with or without tricuspid, MAZE, or LAAC), aortic (incl. reconstruction) valve) and Tumors** **AGE < 75 years** **Sufficient physical condition** Full sternotomy; urgent/complex/redo/prior CS; chest radiation, concomitant bypass Severe comorbidities: arteriopathy, obesity, chronic RI	On-table extubation, PACU to ICU, ward POD 1 Flow of >3.2 L/m^2^ BSA Multidisciplinary Team	**ICU LOS** (hours): 18.5 ± 6 vs. 26.5 ± 29; *p* < 0.01 **Hospital LOS** (days): 6 ± 2 vs. 7 ± 1; *p* < 0.01 **post-op complications** (delirium, AV-Block, ACS, stroke): 14 (13.9%) vs. 20 (18%); *p* = 0.41 - delirium: 5 (5%) vs. 7 (6.3%); *p* = 0.67 New post-op AF: 25 (24.8%) vs. 17 (15.3%); *p* = 0.085 Transfusions necessary: 12 (11.9%) vs. 21 (18.9%); *p* = 0.158 Nosocomial infections: 13 (12.9%) vs. 17 (15.3%); *p* = 0.61 Need for re-exploration: 7 (6.9%) vs. 9 (8.1%); *p* = 0.746 ICU readmission: 4 (4%) vs. 3 (2.7%); *p* = 0.609 Readmission from rehab: 16 (15.8%) vs. 11 (9.9%); *p* = 0.196 CPB time (minutes): 130.5 ± 61 vs. 147 ± 81; *p* = 0.076 XCT (minutes): 77 ± 28 vs. 107 ± 41; *p* < 0.01
Stock et al.—2024 (Augsburg, GER) [16]	Pilot study Deescalating postoperative monitoring: feasibility of 6 h PACU to ward 01/2021–03/2023 ERAS: *n* = 297 Control: *n* = 0 Abort: *n* = 61 (mainly due to prolonged mechanical ventilation)	Age: 63 (55–70)/NA; *p* = NA Sex (M/F): 193/104/NA; *p* = NA BMI: 25 (23–28)/NA; *p* = NA CPB Time (min): NA/NA; *p* = NA	**Age > 18 years** **Deemed suitable for ERAS program** **Marked as ERAS preoperatively** **Successful on-table extubation; hemodynamically stable under low-dose vasopressors** Emergency/redo procedures Neurologic impairment or physical limitation Post-op indication for ICU admission	On table extubation mandatory Looked at whether ICU stay was necessary Major adverse cardiac events	**MACE incidence**: 2 (0.7%) **Bleeding requiring re-exploration**: 4 (1.3%) MACE timing: 5x in ICU; 1x in normal ward **Need for vasoactive drugs** (at 6 h post-op): 30 (10%) **ERAS-associated complications**: 1 (0.3%) reintubation; 2 (0.7%) ICU readmissions
Ertugay et al. 2024 (İzmir, TUR) [17]	Pilot study Developing an ERAS program Total endoscopic mitral surgery program 01/2021–01/2024 ERAS: *n* = 113 Control: *n* = 0 Abort: *n* = 4 (conversion to sternotomy)	Age: 54.7 ± 11.6/NA; *p* = NA Sex (M/F): 51/62/NA; *p* = NA BMI: 25.2 ± 4.2/NA; *p* = NA CPB Time (min): 149.9 ± 30.4/NA; *p* = NA	**Total endoscopic minimally invasive mitral surgery** Conversion to sternotomy, cannulation complications Severe MAC, PAD History of previous thoracotomy Aortic regurgitation > mild Frail status	Peripheral cannulation and 3D endoscope Early catheter removal, early ambulation Discharge on 5 POD	**Bleeding**: 250 mL - re-exploration due to bleeding: 5 (4.4%) **Transfusion rate**: 36 (32%) **Postoperative complications**: - respiratory: 11 (9%) - infectious: 4 3.5% - renal: 4 (3.5%) - mortality: 1 (0.8%) **ICU LOS** (days): 1 (1–18) **Hospital LOS** (days): 5 (3–18)
Pitts et al.—2024 (Berlin, GER) [18]	Propensity-matched analysis No ICU planned after surgery—PACU to ward 01/2021–12/2023 ERAS: *n* = 45 Control: *n* = 90 (after propensity matching)	Age: 55 (46–61)/54 (46–60); *p* = 1.0 Sex (M/F): 39/6/90/12; *p* = 1.0 BMI: 25.0 (22.4–27.1)/24.9 (23.2–27.1); *p* = 0.74 CPB Time (min): 87 (73–108)/94 (77–112); *p* = 0.23	**Age < 80 years** **Euroscore II < 4** LVEF < 35% FEV1 < 50%; OSAS GFR < 50 mL/min or dialysis-dependent chronic RI Neurological impairment (e.g., MRS 3–5) No timely pause of oral anticoagulation (< 24 h)	Mitral valve through lateral (or peri-areolar) mini-thoracotomy; under direct vision or total endoscopic Percutaneous femoral cannulation Endo-aortic clamping under TEE guidance DO2I of ≥280 mL/min/m^2^ MiECC centrifugal pump, retrograde priming)	safety: - **Mortality**: 0 (0%) vs. 0 (0%)); *p* = 1.00 - **Stroke**: 0 (0) vs. 1 (1)); *p* = 0.97 major clinical efficacy: - **Post-op ventilation time**: 140 (100–173) vs. 219 (153–333); *p* = 0.018 - **Hospital LOS** (days): 6 (5–8) vs. 7 (5–9)); *p* = 0.049 Secondary endpoints: - new post-op AF: 4 (9) vs. 8 (9)); *p* = 1.00 - postoperative pain (NRS): 0 (0–4) vs. 3 (1–4)); *p* = 0.005 - need for NIV: 3 (7) vs. 2 (2)); *p* = 0.21 - re-intubation: 1 (2) vs. 0 (0)); *p* = 0.99 - re-exploration for bleeding: 4 (9) vs. 3 (3); *p* = 0.186

Abbreviations: AV = Atrioventricular; BMI = Body Mass Index; BSA = Body Surface Area; CPB = Cardiopulmonary Bypass; CS = Cardiac Surgery; DO2I = Oxygen Delivery Index; ERAS = Enhanced Recovery After Surgery; FEV1 = Forced Expiratory Volume in 1 Second; GDT = Goal-Directed Therapy; GFR = Glomerular Filtration Rate; ICU = Intensive Care Unit; LAAC = Left Atrial Appendage Closure; LVEF = Left Ventricular Ejection Fraction; MAC = Mitral Annular Calcification; MDT = Multidisciplinary Team; MiECC = Minimally Invasive Extracorporeal Circulation; MIVS = Minimally Invasive Valve Surgery; NA = not applicable; NIV = Non-Invasive Ventilation; OSAS = Obstructive Sleep Apnea Syndrome; PACU = Post-Anesthesia Care Unit; PAD = Peripheral Artery Disease; PEEP = Positive End-Expiratory Pressure; POD = Postoperative Day; RI = Renal Insufficiency; TEE = Transesophageal Echocardiography; XCT = Cross-Clamp Time.

**Table 2:** Overview of implemented elements in ERAS programs for MIVS.

ERAS Element	Zaouter et al. [11]	Kubitz et al. [12]/Gebauer et al. [15]	Petersen et al. [13]	Berretta et al. [14]	Stock et al. [16]	Ertugay et al. [17]	Pitts et al. [18]
Shared Decision Making, Patient Engagement, and Education	Meeting with surgeon, physiotherapist, nursing staff, psychologist; video on operating room arrival	Meeting with MDT 2–3 weeks before	Meeting with MDT 2–3 weeks before	Unknown	Unknown	Preoperative education and operative course	ERMICS patient education
Establishment of a Multidisciplinary Team (MDT)	Yes, but MDT not specified	Cardiac surgeon, anesthetist, perfusionist, physiotherapist	Cardiac surgeons, anesthesiologists, cardiologists, perfusionists, physiotherapist	Surgeons, anesthesiologists, perfusionists, physiotherapists, nurses	ERAS nurse (advanced practice nurse), physiotherapist, psychotherapist, anesthesiologist, cardiac surgeon	Cardiac surgeon, nurses, anesthesiologists, perfusionists, dietitians, physiotherapy	ERAS coordinator, MDT (team not specified)
Compliance and Outcomes Auditing	End-of-study monitoring	Pain self-assessments; PONV protocol	Unknown	Unknown	Unknown	Unknown	ERAS coordinator monitoring and troubleshooting
Preoperative Screening and Risk Assessment	Pre-op meeting; screening for tobacco, comorbidities, malnutrition	Frailty scoring; formal physical condition assessment	Frailty scoring; formal physical condition assessment, 6 min walk test	Nutritional correction (if necessary), HbA1c measurement	Individualized risk assessment by senior surgeon	Laboratory analysis, HbA1c, frailty screening	Standardized risk assessment
Prehabilitation	Tailored diet (if necessary)	Daily exercises and nutritional supplementation for 2–3 weeks	Daily exercises and nutritional supplementation for 2–3 weeks	Unknown	Interdisciplinary pre-op clinic visit	Pulmonary/physical rehabilitation, anxiety support, nutritional support	Unknown
Limiting Nil Per Os (NPO) Status	No (Future: carb drink 2 h pre-op, shorten fasting	Maltose carb drink 2 h pre-op	Unknown	NPO after midnight, clear liquid 2–4 h pre-op	Unknown	Unknown	NPO after midnight, clear liquid 2 h pre-op
Transesophageal Echocardiography	Yes	Yes	Unknown	Unknown	Unknown	Yes	Yes
Protective Lung Ventilation	Ventilation on CPB	Unknown	Unknown	Alveolar recruitment by PEEP 10 cm H_2_O	Unknown	Ventilation on CPB	Unknown
Ventilation on Cardiopulmonary Bypass	Tidal volume 3 mL/kg, PEEP 5 cm H_2_O	Unknown	Unknown	Unknown	Unknown	Tidal volume 4–6 mL/kg, PEEP 5–10 cm H_2_O	Unknown
Use of Pulmonary Artery Catheters	Excluded usage	Unknown	Unknown	Unknown	Unknown	Unknown	Unknown
Central Nervous System Monitoring	Unknown	NIRS, bispectral monitoring	NIRS, bispectral monitoring	Unknown	Unknown	NIRS, bispectral monitoring	NIRS, bispectral monitoring
Postoperative Nausea and Vomiting Prevention	Pre-op dexamethasone	Dexamethasone and ondansetron; droperidol (if needed)	Antiemetic prophylaxis (not specified)	Unknown	Dexamethasone; granisetrone and dimenhydrinate	Dexamethasone and ondansetron	Dexamethasone and ondansetron; avoidance of benzodiazepines
Goal-Directed Perfusion	Not in protocol (Future: GDP-strategy on CPB)	Flow > 3.2 L/m^2^ BSA; restrictive vasopressors	Flow > 3.2 L/m^2^ BSA	Unknown	Unknown	Not specified	DO2I ≥ 280 mL/min/m^2^, hypothermia
Early Extubation Strategies/IntraOP Extubation	Remifentanil use; extubation criteria met in ICU	Early NIV, on-table extubation	On-table extubation	On-table extubation	On-table extubation; no NIV	Extubation within 6 h post-op	Remifentanil use; extubation after normothermia
Aki Prevention and Management	Fluid loading; vena cava variability monitoring	Unknown	Furosemide, CPB hypothermia	Use of biomarkers	Unknown	Unknown	DO2I ≥ 280 mL/min/m^2^, hypothermia
Postoperative Activity and Sternal Precautions	Sitting in chair after 4 h	first physio after 2–3 h; extended physio afterward	first physio after 2–3 h; second physio in the evening by nursing, extended physio afterward	respiratory therapy after 3–6 h after; early mobilization after 6–12 h	Unknown	Sitting outside bed morning; ambulation evening POD 1	Physio in PACU to bedside, sometimes standing; respiratory; individualized physiotherapy
Goal-Directed Therapy (GDT)	GDT algorithm	Restrictive fluid therapy; hemofiltration on CPB	Restrictive fluid therapy	GDT with fluids, vasopressors, inotropes (not specified)	Unknown	Vasopressors vs. fluids (not specified)	GDT by ERAS anesthesiologist
Opioid-Sparing Pain Management	Multimodal: up to 8 agents; acetaminophen, nefopam, magnesium, pregabalin	Focus on PACU: metamizole and piritramide	Unknown	No (morphine and tramadol infusion)	Structured tapering; POD 3 opioid cessation	Escalating analgesic regimen: acetaminophen, dextromethorphan, tramadol	Structured tapering; opioid cessation after drain removal
Regional Analgesia	Wound infiltration (ropivacaine 0.75%)	Intercostal catheter + ropivacaine	Regional anesthesia (not further specified)	Serratus block, lidocaine-ropivacaine infiltration	Parasternal/serratus block pre-surgery	Cryotherapy, local neuroblocker/perfusion catheter	Serratus block at surgery end (ropivacaine 0.375%)
Patient Blood Management Program	Transfusion trigger Hb < 7.2	Transfusion trigger Hb < 7.5	Unknown	MiECC	Unknown	Anemia diagnostics (iron supplementation if necessary), retrograde priming, normovolemic hemodilution, antifibrinolytics	MiECC, Retrograde priming
Postoperative Atrial Fibrillation Prevention	Unknown	Amiodarone infusion (for high-risk patients), AF ablation, LAAC	Low-dose amiodarone prophylaxis	Unknown	Unknown	AF ablation, LAAC	AF ablation, LAAC
Systematic Delirium Screening and Prevention	Benzodiazepines avoided pre-op; AGS statement followed	Unknown	Unknown	Early family contact, delirium screening 1x/shift	Unknown	Alcohol cessation focus	Early family contact (in-person or video)
Surgical Site Infection Prevention Bundle	Unknown	Normothermia post-op	Unknown	Glycemic control, antibiotics, smoking cessation	Glycemic control, antibiotics	Glycemic control, antibiotics	Glycemic control, antibiotics

Abbreviations: AF = Atrial Fibrillation; AGS = American Geriatric Society; BSA = Body Surface Area; CPB = Cardiopulmonary Bypass; DO2I = Oxygen Delivery Index; ERAS = Enhanced Recovery After Surgery; GDT = Goal-Directed Therapy; HbA1c = Hemoglobin A1c; ICU = Intensive Care Unit; LAAC = Left Atrial Appendage Closure; MDT = Multidisciplinary Team; MiECC = Minimally Invasive Extracorporeal Circulation; MIVS = Minimally Invasive Valve Surgery; NIRS = Near-Infrared Spectroscopy; NIV = Non-Invasive Ventilation; NPO = Nil Per Os; PACU = Post-Anesthesia Care Unit; PEEP = Positive End-Expiratory Pressure; POD = Postoperative Day; PONV = Postoperative Nausea and Vomiting; TEE = Transesophageal Echocardiography.

**Table 3:** Comparison of ERAS elements implemented across studies.

Category	Element	Zaouter et al. [11]	Kubitz et al. [12]/Gebauer et al. [15]	Petersen et al. [13]	Berretta et al. [14]	Stock et al. [16]	Ertugay et al. [17]	Pitts et al. [18]	QoE
General	1. Shared Decision Making, Patient Engagement	Y	Y	Y	N	N	Y	Y	Low
	2. Establishment of a Multidisciplinary Team	Y	Y	Y	Y	Y	Y	Y	Moderate
	3. Compliance and Outcomes Auditing	Y	Y	N	N	N	N	Y	Moderate
Pre-OP	4. Preoperative Screening and Risk Assessment	Y	Y	Y	Y	Y	Y	Y	Moderate
	5. Prehabilitation	Y	Y	Y	N	Y	Y	N	Low
	6. Limiting Nil Per Os Status	N	Y	N	Y	N	N	Y	Low
Intraoperative	7. Transesophageal Echocardiography	Y	Y	N	N	N	Y	Y	Moderate
	8. Protective Lung Ventilation	Y *	N	N	Y	N	Y *	N	High
	9. Ventilation on Cardiopulmonary Bypass	Y	N	N	N	N	Y	N	Moderate
	10. Use of Pulmonary Artery Catheters (excluded use)	Y	N	N	N	N	N	N	Moderate
	11. Central Nervous System Monitoring	N	Y	Y	N	N	Y	Y	Moderate
	12. Postoperative Nausea and Vomiting Prevention	Y	Y	Y	N	Y	Y	Y	Moderate
	13. Goal-Directed Perfusion	N	Y	Y	N	N	N/S	Y	Low
Post-OP	14. Early Extubation Strategies	Y	Y	Y	Y	Y	Y	Y	Moderate
	15. Intraoperative Extubation	Y	Y	Y	Y	Y	N	N	Low
	16. AKI Prevention and Management	Y	N	Y	Y	N	N	Y	Moderate
	17. Postoperative Activity and Sternal Precautions	Y	Y	Y	Y	N	Y	Y	Moderate
Multiphase	18. Goal-Directed Therapy	Y	Y	Y	Y	N	N/S	N/S	Moderate
	19. Opioid-Sparing Pain Management	Y	N/S	N	N	Y	Y	Y	Moderate
	20. Regional Analgesia	Y	Y	Y	Y	Y	Y	Y	Moderate
	21. Patient Blood Management Program	Y	Y	N	Y	N	Y	Y	Moderate
	22. Postoperative Atrial Fibrillation Prevention	N	Y	Y	N	N	Y	Y	Moderate
	23. Systematic Delirium Screening and Prevention	Y	N	N	Y	N	Y	Y	High
	24. Surgical Site Infection Prevention Bundle	N	Y	N	Y	Y	Y	Y	High
	Total elements implemented (out of 24 elements)	18	18	14	12	9	16	18	

Abbreviations: Y = yes; N/S = not specified/not enough detail; N = not in protocol/unknown; * = counted as not enough detail as changes are for a different ERAS element. Y/green: was used and counted; Y */yellow was used but only indirectly and therefore not counted as it was mainly part of different measure and was not sufficiently addressed; N/S/yellow was somehow named in original trial, but not described and could therefore not be counted; N/red: was not named/addressed and was therefore not counted to implemented elements

## Data Availability

The original contributions presented in this study are included in the article. Further inquiries can be directed to the corresponding author.

## Abbreviations

AKI	Acute Kidney Injury
CPB	Cardiopulmonary Bypass
DO2I	Oxygen Delivery Index
ERAS	Enhanced Recovery After Surgery
GDT	Goal-Directed Therapy
ICU	Intensive Care Unit
MDT	Multidisciplinary Team
MiECC	Minimally Invasive Extracorporeal Circulation
MIVS	Minimally Invasive Valve Surgery
PACU	Post-Anesthesia Care Unit
PEEP	Positive End-Expiratory Pressure
PONV	Postoperative Nausea and Vomiting
SSI	Surgical Site Infection
TEE	Transesophageal Echocardiography
